# Efficacy and safety of sodium zirconium cyclosilicate in patients with baseline serum potassium level ≥ 5.5 mmol/L: pooled analysis from two phase 3 trials

**DOI:** 10.1186/s12882-019-1611-8

**Published:** 2019-12-02

**Authors:** Alpesh N. Amin, Jose Menoyo, Bhupinder Singh, Christopher S. Kim

**Affiliations:** 1 Present Address: University of California Irvine Medical Center, 101 The City Drive South, Orange, CA 92868 USA; 2 Affiliation at the time the study was conducted: ZS Pharma, Inc., a member of the AstraZeneca family of companies, San Mateo, CA USA; 3 Division of General Medicine, University of Washington, Seattle, WA USA

**Keywords:** Efficacy, Hyperkalemia, Safety, Sodium zirconium cyclosilicate

## Abstract

**Background:**

Reliable, timely-onset, oral treatments with an acceptable safety profile for patients with hyperkalemia are needed. We examined the efficacy and safety of sodium zirconium cyclosilicate (SZC; formerly ZS-9) treatment for ≤ 48 h in patients with baseline serum potassium level ≥ 5.5 mmol/L.

**Methods:**

Data were pooled from two phase 3 studies (ZS-003 and HARMONIZE) among patients receiving SZC 10 g three times daily. Outcomes included mean and absolute change from baseline, median time to potassium level ≤ 5.5 and ≤ 5.0 mmol/L, and proportion achieving potassium level ≤ 5.5 and ≤ 5.0 mmol/L at 4, 24, and 48 h. Outcomes were stratified by baseline potassium. Safety outcomes were evaluated.

**Results:**

At baseline, 125 of 170 patients (73.5%) had potassium level 5.5–< 6.0, 39 (22.9%) had potassium level 6.0–6.5, and 6 (3.5%) had potassium level > 6.5 mmol/L. Regardless of baseline potassium, mean potassium decreased at 1 h post-initial dose. By 4 and 48 h, 37.5% and 85.0% of patients achieved potassium level ≤ 5.0 mmol/L, respectively. Median (95% confidence interval) times to potassium level ≤ 5.5 and ≤ 5.0 mmol/L were 2.0 (1.1–2.0) and 21.6 (4.1–22.4) h, respectively. Fifteen patients (8.8%) experienced adverse events; none were serious.

**Conclusions:**

SZC 10 g three times daily achieved serum potassium reduction and normokalemia, with a favorable safety profile.

**Trial registration:**

ClinicalTrials.gov identifiers: ZS-003: NCT01737697 and HARMONIZE: NCT02088073.

## Background

Hyperkalemia is a potentially life-threatening electrolyte abnormality associated with poor clinical outcomes [1], cardiac dysrhythmias [2], increased mortality [3–5], and higher use of healthcare resources [1]. Despite the potential for devastating sequelae, the threshold for and means of treating hyperkalemia vary in clinical practice [6].

Hyperkalemia therapies (e.g., intravenous insulin and glucose or inhaled β2 agonists) typically used in the emergency department and other acute care settings to lower serum potassium (K^+^) are temporary in nature and impractical for ongoing outpatient use [6, 7]. Although acute dialysis may be used in select patients for the immediate treatment of hyperkalemia, this procedure is invasive and costly, presents logistical challenges, and may require an inpatient admission [7]. While nonspecific cation-exchange resins (e.g., sodium polystyrene sulfonate [SPS] and patiromer) are adequately suited for the outpatient setting, they have been associated with questionable tolerability [8, 9] or a slow onset of action [6, 8, 10]. Therefore, there remains a need for more reliable treatment options for patients with hyperkalemia that allow for rapid and safe reduction of K^+^ values.

Sodium zirconium cyclosilicate (SZC; formerly ZS-9) is an oral, nonabsorbed, high-capacity cation-binding compound that selectively exchanges K^+^ for hydrogen and sodium ions throughout the gastrointestinal tract [11]. SZC is thought to start binding potassium in the upper gastrointestinal tract, which most likely accounts for its rapid onset of action [11, 12] (AstraZeneca, unpublished observations). In clinical studies of patients with hyperkalemia, SZC has been shown to reduce serum K^+^ levels within 1 h of administration [7, 13–15]. SZC is currently approved for the treatment of patients with hyperkalemia in the United States (US) and the European Union. The current analysis examined the efficacy and safety of SZC for up to 48 h among patients with moderate to severe hyperkalemia (baseline serum K^+^ level ≥ 5.5 mmol/L). These results may have potential implications for treating patients with moderate to severe hyperkalemia that is not immediately life-threatening.

## Materials and methods

### Design and setting

This post hoc analysis included data pooled from two published, randomized, double-blind, phase 3 trials: ZS-003 (ClinicalTrials.gov identifier: NCT01737697) and HARMONIZE (NCT02088073). Primary safety and efficacy data from both studies previously have been reported [13, 15], and short-term findings of a subset of data from this post hoc analysis previously have been summarized [7]. Briefly, both trials enrolled patients to receive SZC (ZS-003 also included a placebo arm) three times daily (TID) for up to 48 h during the correction phase, followed by once-daily maintenance dosing for 12 days (ZS-003) or 28 days (HARMONIZE) in an outpatient setting. Both phase 3 studies were conducted in accordance with Good Clinical Practice guidelines and the provisions of the Declaration of Helsinki, and all patients provided written informed consent before study participation.

### Study population

Inclusion criteria for the pivotal phase 3 studies included ambulatory patients aged ≥ 18 years. Patients were excluded from the studies if they were being treated as an inpatient, undergoing dialysis, had a life expectancy of < 3 months, experienced cardiac dysrhythmia that required immediate treatment, or had received treatment with K^+^-binding resins within 7 days. Patients selected for this analysis had baseline serum K^+^ levels ≥ 5.5 mmol/L and received SZC 10 g TID for up to 48 h in the correction phase of either study. Patients were excluded from the analysis if they had a baseline serum K^+^ level < 5.5 mmol/L or had received any other dose of SZC. The analysis population was further stratified by baseline serum K^+^ level (5.5–< 6.0, 6.0–6.5, and > 6.5 mmol/L).

### Interventions and study outcomes

All patients received SZC 10 g TID with meals for 48 h. SZC was administered as a tasteless, odorless, white powder mixed with water. On study day 1, SZC was administered before breakfast, and with meals for the remainder of both studies. Patients were maintained on their usual medications throughout each study, including diuretic agents, renin–angiotensin–aldosterone system inhibitor (RAASi) therapies, and glucose-lowering treatments. Patients were not subjected to dietary restrictions and were instructed to maintain their usual diet without specified alterations.

In both trials, K^+^ levels were measured at 0, 1, 2, 4, 24, 25, and 48 h following the first dose of SZC, with the 0-, 1-, and 2-h measurements occurring before breakfast on day 1. K^+^ levels were measured through point-of-care testing using an i-STAT device (Abbott Point of Care, Inc.; Princeton, NJ, USA) for immediate treatment decisions and via the central laboratory (referred to as serum K^+^) for the assessment of primary and secondary outcomes, as previously described [13, 15]. Outcomes of interest for the current analysis included mean serum and i-STAT K^+^ levels over time; absolute change from baseline in serum and i-STAT K^+^ levels; median time to serum and i-STAT K^+^ levels of ≤ 5.5, ≤ 5.1, and ≤ 5.0 mmol/L; proportions of patients achieving serum and i-STAT K^+^ levels of ≤ 5.5, ≤ 5.1, and ≤ 5.0 mmol/L at 4, 24, and 48 h; and safety data (including adverse events [AEs] and serum laboratory data).

### Statistical analysis

Descriptive statistics were provided for all categorical outcomes. Statistical analyses were based on serum K^+^ levels as measured by the central laboratory. Paired *t* tests were used to evaluate the change from baseline in serum K^+^ and laboratory parameters. Kaplan-Meier life tables were used to estimate the time to serum K^+^ and i-STAT K^+^ levels of ≤ 5.5, ≤ 5.1, and ≤ 5.0 mmol/L and are presented as median ± 95% confidence interval (CI). The 95% CI for the median time to normalization was calculated with distribution-free assumption nonparametric method. Patients who did not meet the K^+^ level goals were censored for the analysis on the last treatment day during the correction phase. For any missing time measurement from the database for analyses of i-STAT K^+^ time to achievement of K^+^ goals, the nominal time point for that patient was used for calculations. Safety data were summarized as incidence (number and percentage of patients).

## Results

### Baseline demographics and characteristics

Of the 401 patients who received SZC 10 g TID for up to 48 h during the correction phase of the two phase 3 studies, 170 (42.4%) had a baseline serum K^+^ level of ≥ 5.5 mmol/L. Baseline demographic and clinical data are summarized in Table 1. Most patients were male (61.8%), with a mean (standard deviation) age of 65.7 (12.1) years. There was a high burden of comorbidities in this patient population, including chronic kidney disease, congestive heart failure, and diabetes mellitus. Nearly all patients were taking concomitant medications at baseline (98.2%). The most common medications were RAASi therapies (66.5%), followed by diuretics (45.9%) and β-blockers (45.3%). The mean (standard deviation) number of medications per patient was 8.7 (4.5).

**Table 1:** Baseline demographics and characteristics of patients with baseline serum K^+^ ≥ 5.5 mmol/L

Parameter	Overall (*N* = 170)
Age, years, mean (SD)	65.7 (12.1)
Male, n (%)	105 (61.8)
Race, n (%)	
White	134 (78.8)
Black or African American	31 (18.2)
Asian	3 (1.8)
Other	2 (1.2)
Ethnicity, n (%)	
Hispanic	44 (25.9)
Non-Hispanic	126 (74.1)
Serum K^+^, mmol/L, n (%)	170 (100.0)
5.5 to < 6.0	125 (73.5)
6.0 to 6.5	39 (22.9)
> 6.5	6 (3.5)
Serum K^+^, mmol/L, mean (95% CI)	5.8 (5.8–5.9)
5.5 to < 6.0	5.7 (5.7–5.7)
6.0 to 6.5	6.2 (6.1–6.2)
> 6.5	6.9 (6.6–7.2)
eGFR, mL/min/1.73 m^2^, n (%)	
< 15	28 (16.5)
15 to < 30	55 (32.4)
30 to < 60	51 (30.0)
≥ 60	32 (18.8)
Missing	4 (2.4)
Comorbidity, n (%)^a^	
Chronic kidney disease	113 (66.5)
Diabetes mellitus	112 (65.9)
Heart failure	22 (12.9)
Concomitant medications, n (%)	167 (98.2)
RAASi	113 (66.5)
Diuretics	78 (45.9)
β-blockers	77 (45.3)
Medications received, mean (SD)	8.7 (4.5)

*CI* confidence interval, *eGFR* estimated glomerular filtration rate, *K*^*+*^ potassium, *RAASi* renin–angiotensin–aldosterone system inhibitor, *SD* standard deviation

^a^As defined by standardized Medical Dictionary for Regulatory Activities query narrow terms

Of the 170 patients with a baseline serum K^+^ level ≥ 5.5 mmol/L included in this analysis, the mean serum K^+^ level at baseline was 5.83 (95% CI, 5.78–5.88) mmol/L. Most patients (*n* = 125 [73.5%]) had a baseline serum K^+^ level 5.5–< 6.0 mmol/L, with a mean serum K^+^ level of 5.68 (95% CI, 5.65–5.70) mmol/L. Thirty-nine patients had a baseline serum K^+^ level of 6.0–6.5 mmol/L (mean [95% CI], 6.18 [6.13–6.24] mmol/L), and 6 patients had a baseline serum K^+^ level of > 6.5 mmol/L (mean [95% CI], 6.87 [6.59–7.15] mmol/L).

### Efficacy outcomes

**Fig. 1:**
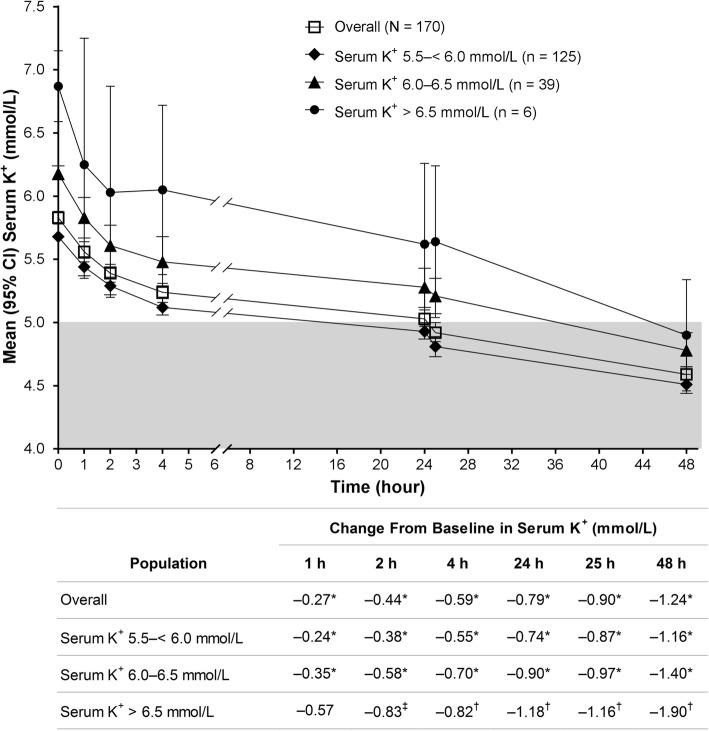
Mean (95% CI) serum K^+^ over time. CI, confidence interval; K^+^, potassium. *p ≤ 0.001; ^†^*p* < 0.01; ^‡^*p* < 0.05 for absolute change from baseline

Treatment with SZC 10 g TID in the correction phase resulted in a reduction from baseline in mean serum K^+^ levels in the overall population and in all serum K^+^ subgroups (Fig. 1). In the overall population, serum K^+^ levels decreased as early as 1 h after the first dose of SZC 10 g (mean [95% CI], −0.27 [−0.34 to −0.21]; *p* < 0.001 vs. baseline). Similar results were observed in the 5.5–< 6.0 and 6.0–6.5 mmol/L subgroups (*p* ≤ 0.001 for all comparisons vs. baseline). Patients in the > 6.5 mmol/L subgroup experienced a change from baseline in serum K^+^ levels of −0.83 mmol/L by 2 h post dose (*p* < 0.05 vs. baseline). By 4 h post dose, changes from baseline in serum K^+^ levels of −0.55, −0.70, and −0.82 mmol/L were observed in the 5.5–< 6.0, 6.0–6.5, and > 6.5 mmol/L subgroups, respectively (*p* ≤ 0.01 for all comparisons vs. baseline). Mean serum K^+^ levels were decreased by 48 h after the initial dose in all baseline K^+^ strata (Fig. 1). Patients with baseline serum K^+^ level > 6.5 mmol/L experienced the largest reduction in serum K^+^ levels by 48 h (−1.90 mmol/L; *p* < 0.01 vs. baseline). Similar results were observed when i-STAT K^+^ levels were examined over time in the overall population and in all baseline serum K^+^ strata (Additional file 1: Figure S1).

**Fig. 2:**
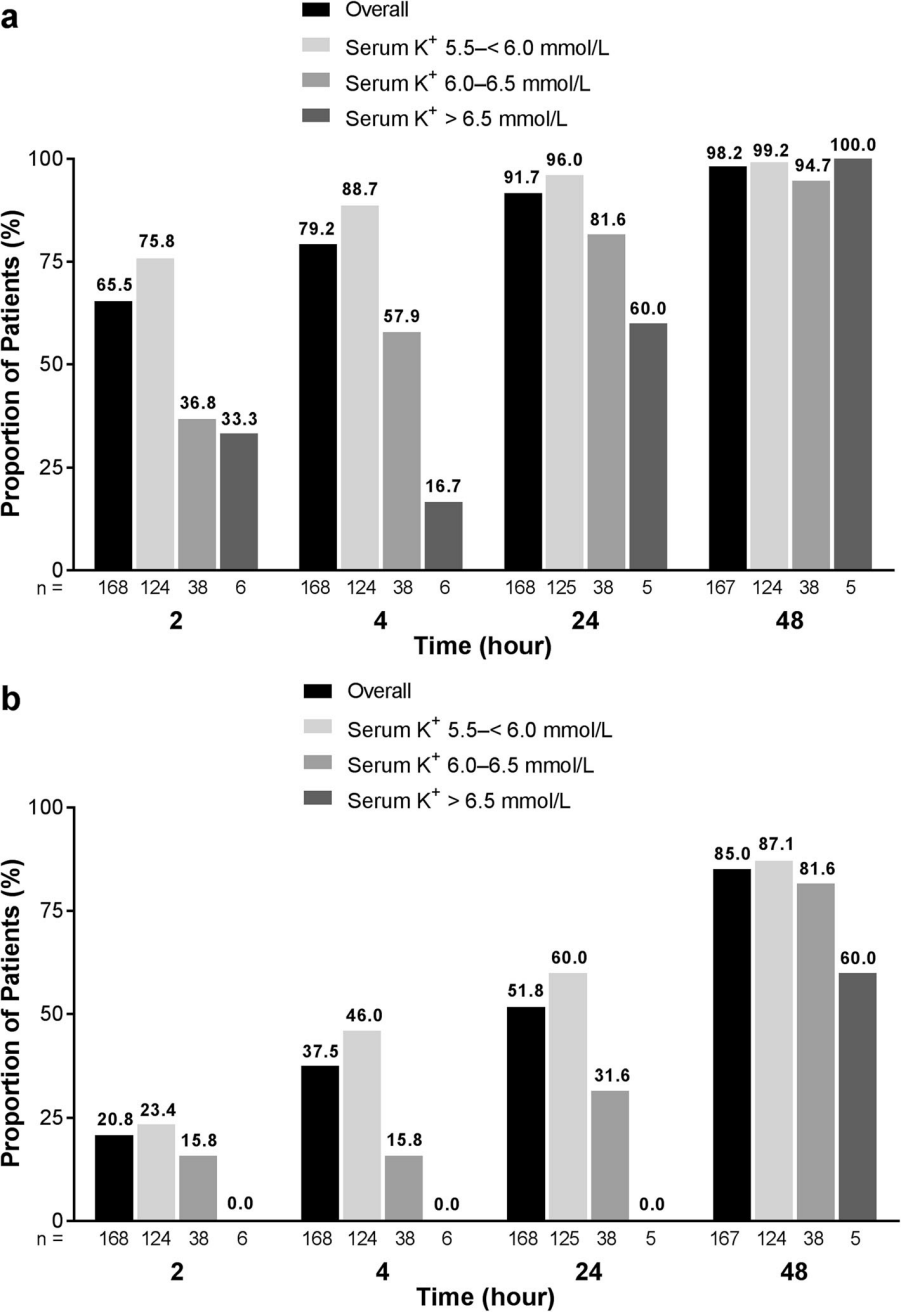
Patients with serum potassium (K^+^) levels (**a**) ≤ 5.5 mmol/L and (**b**) ≤ 5.0 mmol/L at various time points

A serum K^+^ level of ≤ 5.5 mmol/L was achieved by 66% of patients within 2 h of initial treatment with SZC 10 g, and nearly 80 and 98% of patients achieved this serum K^+^ level within 4 and 48 h, respectively (Fig. 2a). A serum K^+^ level of ≤ 5.0 mmol/L was achieved by more than one-third (38%) of patients by 4 h after the initial dose of SZC 10 g, and 85% of patients achieved this serum K^+^ level at 48 h (Fig. 2b). Data for the proportions of patients who achieved a serum K^+^ level of ≤ 5.1 mmol/L are presented in Additional file 2: Figure S2. In general, similar proportions of patients achieved i-STAT K^+^ goals over time in the overall population and in all baseline serum K^+^ strata (Additional file 3: Figure S3).

The median time to a serum K^+^ level of ≤ 5.5 mmol/L in the overall population was 2.0 (95% CI, 1.1–2.0) h (Table 2). The median time to normalization of serum K^+^ levels to ≤ 5.0 mmol/L was 21.6 (95% CI, 4.1–22.4) h. Although patients with higher baseline serum K^+^ levels experienced longer median times to normalization than patients with lower serum K^+^ levels, all but one patient with baseline serum K^+^ level of > 6.5 mmol/L achieved serum K^+^ ≤ 5.5 mmol/L within 48 h (median time [95% CI], 20.6 [2.2–24.3] h). The median time to achievement of serum K^+^ level ≤ 5.1 mmol/L is presented in Additional file 4: Table S1. When examining the time to achievement of i-STAT K^+^ levels ≤ 5.5, ≤ 5.1, and ≤ 5.0 mmol/L, it was found that i-STAT K^+^ goals were achieved in less time than serum K^+^ goals (Additional file 5: Table S2).

**Table 2:** Median time to achievement of serum K^+^ levels ≤ 5.5 and ≤ 5.0 mmol/L in the correction phase

Median (95% CI) time to serum K^+^ level, hours	Overall (*N* = 170)	Baseline Serum K^+^ level (mmol/L)		
		5.5–< 6.0 (*n* = 125)	6.0–6.5 (*n* = 39)	> 6.5 (*n* = 6)
Serum K^+^ ≤ 5.5 mmol/L	1.97 (1.08–2.00)	1.08 (1.03–1.97)	3.97 (2.00–19.63)	20.55 (1.00–47.97)
Serum K^+^ ≤ 5.0 mmol/L	21.55 (4.08–22.42)	4.22 (4.00–21.92)	23.50 (20.85–45.27)	45.30 (1.00–48.70)

*CI* confidence interval, *K*^*+*^ potassium

### Safety

AEs were observed in 15 patients (8.8%) with baseline serum K^+^ level of ≥ 5.5 mmol/L during the first 48 h of treatment (Table 3). Most AEs were gastrointestinal-related disorders (5 patients [2.9%]). No serious AEs were reported during the correction phase.

**Table 3:** AEs and SAEs in patients who received SZC 10 g in the pooled correction phase

Parameter, n (%)	Overall (*N* = 170)	Baseline Serum K^+^ level (mmol/L)		
		5.5–< 6.0 (*n* = 125)	6.0–6.5 (*n* = 39)	> 6.5 (*n* = 6)
Any AEs^a^	15 (8.8)	7 (5.6)	8 (20.5)	0 (0.0)
Gastrointestinal disorders	5 (2.9)	2 (1.6)	3 (7.7)	0 (0.0)
Constipation	3 (1.8)	1 (0.8)	2 (5.1)	0 (0.0)
Diarrhea	1 (0.6)	0 (0.0)	1 (2.6)	0 (0.0)
Peripheral edema	1 (0.6)	0 (0.0)	1 (2.6)	0 (0.0)
Upper respiratory tract infection	1 (0.6)	0 (0.0)	1 (2.6)	0 (0.0)
Blood glucose decreased	1 (0.6)	0 (0.0)	1 (2.6)	0 (0.0)
Headache	1 (0.6)	0 (0.0)	1 (2.6)	0 (0.0)
Cough	1 (0.6)	0 (0.0)	1 (2.6)	0 (0.0)
Ecchymosis	1 (0.6)	0 (0.0)	1 (2.6)	0 (0.0)
Serious AEs	0 (0.0)	0 (0.0)	0 (0.0)	0 (0.0)

*AE* adverse event, *K*^***+***^ potassium, *SAE* serious adverse event, *SZC* sodium zirconium cyclosilicate

^a^Any AE that occurred in > 2% of patients, regardless of relation to study drug. Bold text indicates total number of events

At 48 h, no cases of hypokalemia (serum K^+^ level < 3.5 mmol/L), 1 case (0.6%) of hypocalcemia (< 1.8 mmol/L), and 3 new cases (27.3%) of hypomagnesemia (< 0.6 mmol/L) were observed. Changes from baseline to 48 h were observed for mean magnesium, calcium, and bicarbonate (Additional file 6: Table S3).

## Discussion

This post hoc analysis demonstrated that SZC reduces serum K^+^ levels in patients with baseline serum K^+^ levels of ≥ 5.5 mmol/L. Furthermore, in this patient population, a single treatment with SZC 10 g reduced serum K^+^ levels at 1 h after the initial dose. Treatment with SZC 10 g TID resulted in a continued and sustained reduction in serum K^+^ levels for up to 48 h after the initial dose. These trends held true for all baseline serum K^+^ strata. In addition, SZC 10 g demonstrated a safety profile consistent with that observed in prior studies [13, 15].

A single-dose treatment with SZC 10 g reduced mean serum K^+^ levels within 1 h of the initial dose by 0.27 mmol/L in patients with a baseline serum K^+^ level ≥ 5.5 mmol/L. In general, the most marked reductions in serum K^+^ levels were observed in patients with the highest serum K^+^ level at baseline in previous studies [13], which was confirmed in the present analysis. Nearly two-thirds (65.5%) of all patients with moderate to severe hyperkalemia achieved serum K^+^ level ≤ 5.1 mmol/L within 24 h in the present study, which was achieved by 84% of the overall patient population in the same time period in the HARMONIZE trial [13]. This trend was further reflected in the median time to serum K^+^ level ≤ 5.0 mmol/L, where patients in the overall population of the HARMONIZE trial demonstrated normalized serum K^+^ levels in 2.2 h [13]. This corresponded with a median time of 21.6 h in the current analysis, which is expected for a patient population with a higher mean baseline serum K^+^ level than that observed in the HARMONIZE trial.

In the current analysis, a single dose of SZC 10 g resulted in most patients (79%) with a baseline K^+^ level ≥ 5.5 mmol/L achieving a serum K^+^ level ≤ 5.5 mmol/L, and more than one-third (38%) of all patients achieving a K^+^ level ≤ 5.0 mmol/L within 4 h. These results are consistent with those from an earlier pooled analysis of the same two trials assessing patients with a slightly higher baseline serum K^+^ threshold (≥ 6.0 mmol/L), which found that 52% of patients achieved a serum K^+^ level ≤ 5.5 mmol/L by 4 h after the first SZC 10 g dose [7]. Considering the mean baseline serum K^+^ level in the previous analysis was 6.3 mmol/L, it is not unexpected that fewer patients in that analysis were able to achieve normalization rates comparable to that seen in the current analysis at 4 h.

The safety profile of SZC 10 g TID demonstrated in the current patient population was generally consistent with that observed in the overall populations who received SZC during the two phase 3 trials [13, 15]. The most common AEs experienced by patients in this analysis were gastrointestinal-related disorders (2.9%), with constipation being the most frequent gastrointestinal AE (1.8%). In the HARMONIZE study, the most common AE was edema [12], and in the ZS-003 study, the most common AE in both placebo and SZC treatment groups was diarrhea [14].

The clinical and economic burden of hyperkalemia is well known. In a recent analysis of the cost of hyperkalemia in the United States (*N* = 39,626 patients with hyperkalemia and an equal number of matched controls), patients with hyperkalemia incurred $4128 higher 30-day healthcare costs than matched controls ($5994 vs. $1866, respectively) [16]. A review of 911,698 medical records across the US demonstrated that, in the absence of known cardiovascular disease, diabetes, heart failure, or chronic kidney disease, mortality was shown to be 7.5-fold higher in individuals with K^+^ levels of 5.5–8.0 mmol/L vs. those with K^+^ levels of 4.0–< 5.0 mmol/L [17]. In a cohort study of more than 55,000 patients with chronic kidney disease not undergoing dialysis, serum K^+^ levels of ≥ 6.0 mmol/L were associated with a higher risk of death, major adverse cardiovascular events, hospitalizations, and discontinuations of RAASi therapy [1]. Elevated K^+^ was the cause of approximately 67,000 visits to US emergency departments during 2011, with 50% of these visits resulting in hospital admissions [18]. The average length of stay associated with a hyperkalemia admission was 3.2 days, with an estimated cost of $24,178. Among Medicare beneficiaries, total annual hospital expenditures for admissions with hyperkalemia as the primary diagnosis totaled nearly $700 million [18]. More recent US data in 2014 demonstrated that patients admitted with a primary diagnosis of hyperkalemia accrued an average length of stay of 3.3 days and a mean cost of $29,181 per stay, for an estimated total annual hospital charge of $1.2 billion [19]. New hyperkalemia treatment options may have the potential to positively impact healthcare resource utilization.

Orally available therapeutic options for hyperkalemia, including cation-binding compounds to eliminate K^+^, have been in use since the late 1950s [20]. SPS and patiromer are the only other K^+^-binding resins currently approved for clinical use in the United States. However, there is limited evidence to support their efficacy and safety in the acute treatment of patients with serum K^+^ level ≥ 5.5 mmol/L. SPS was approved for use in the United States in the absence of robust clinical trial evidence to support drug efficacy [20, 21]. SPS has a slow onset of action [10, 20] and poor predictability in its ability to decrease serum K^+^ levels, and is, therefore, not indicated for the acute treatment of severe hyperkalemia [22]. In addition, SPS and patiromer have been associated with gastrointestinal-related AEs [8, 22–24].

There are several limitations of this study. This post hoc analysis lacked a placebo control and included a limited number of patients with a serum K^+^ level ≥ 6.0 mmol/L (*n* = 45). The phase 3 studies were performed in an outpatient population, which may have limited the number of patients with more severe hyperkalemia. The current analysis focused only on the correction phase of treatment. While both phase 3 studies included maintenance-phase treatment periods, those data could not be pooled for additional analysis because of differences in study designs. Further studies in the acute setting (e.g. emergency department and the hospital), in patients undergoing dialysis, and those that evaluate the impact of treatment on readmission rates are merited. Additional studies that evaluate follow-up care and the ability to safely maintain K^+^ values after acute care management are also warranted. Given the mechanism of action of SZC, we do not expect a difference in the treatment response between men and women. Further analysis would need to be carried out to verify this more formally.

## Conclusions

Patients with baseline serum K^+^ level ≥ 5.5 mmol/L who received SZC 10 g experienced decreases in serum K^+^ levels from baseline within 1 h of treatment, and most patients (85%) who received SZC 10 g TID achieved normokalemia within 48 h. The reduction of serum K^+^ levels among patients with serum K^+^ level ≥ 5.5 mmol/L with SZC was achieved with an acceptable safety profile, consistent with that observed in prior studies. These results may be of particular interest to hospitalists, observation/short-stay unit providers, and urgent care and emergency department providers, who may see patients with hyperkalemia and are uncertain about their clinical trajectory. These patients are currently observed in these settings for variable periods of time at a high cost, and the results of this analysis may help guide the treatment and transition plans for these patients.

## Supplementary information

**Additional file 1: Figure S1.** Mean (95% CI) i-STAT K^+^ over time in patients with baseline serum K^+^ level ≥ 5.5 mmol/L.

**Additional file 2: Figure S2.** Proportions of patients who achieved serum potassium (K^+^) levels of ≤ 5.1 mmol/L at 4, 24, and 48 h.

**Additional file 3: Figure S3.** Proportions of patients who achieved i-STAT K^+^ levels of ≤ 5.5 (A), ≤ 5.1 (B), and ≤ 5.0 mmol/L (C) in patients with baseline serum K^+^ level ≥ 5.5 mmol/L at 4, 24, and 48 h.

**Additional file 4: Table S1.** Median time to achievement of serum K^+^ level ≤ 5.1 mmol/L in the correction phase.

**Additional file 5: Table S2.** Median time to achievement of i-STAT K^+^ levels ≤ 5.5, ≤ 5.1, and ≤ 5.0 mmol/L in patients with baseline serum K^+^ level ≥ 5.5 mmol/L in the correction phase.

**Additional file 6: Table S3.** Serum chemistry laboratory parameters in the correction phase (*N* = 166).

## Data Availability

Data supporting the conclusions of this article may be obtained in accordance with AstraZeneca’s data sharing policy described at https://astrazenecagrouptrials.pharmacm.com/ST/Submission/Disclosure.

## Abbreviations

AE: Adverse event
CI: Confidence interval
K^+^: Potassium
RAASi: Renin–angiotensin–aldosterone system inhibitor
SPS: Sodium polystyrene sulfonate
SZC: Sodium zirconium cyclosilicate
TID: Three times daily
US: United States
