# Supplementary figures and images for: Efficacy and safety of sodium zirconium cyclosilicate in patients with baseline serum potassium level ≥ 5.5 mmol/L: pooled analysis from two phase 3 trials
Source: BMC Nephrol. 2019 Dec 2;20:440. doi: 10.1186/s12882-019-1611-8 (PMC6889520; doi:10.1186/s12882-019-1611-8)

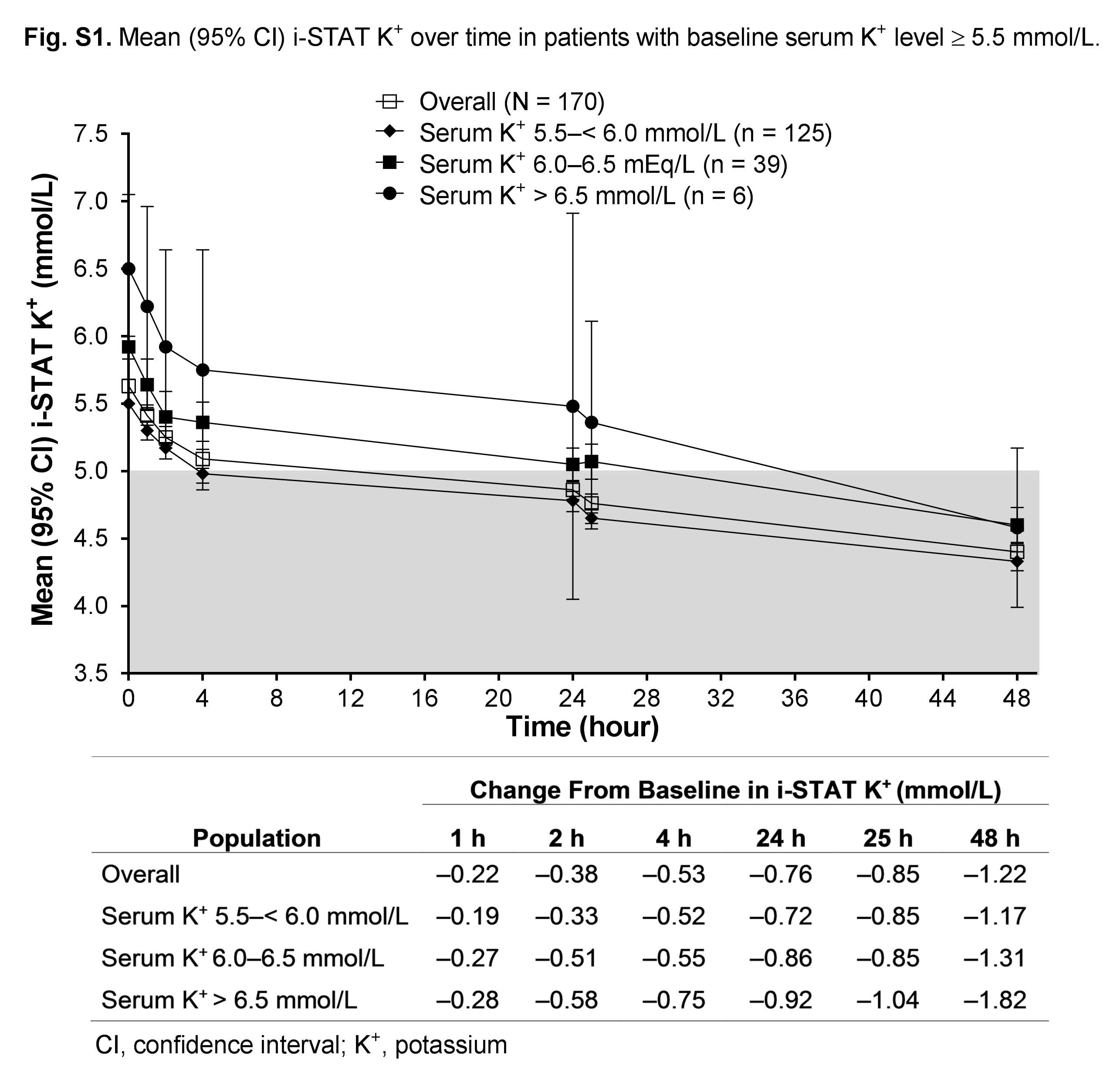

Supplement: Supplementary file 1 — Additional file 1: Figure S1. Mean (95% CI) i-STAT K+ over time in patients with baseline serum K+ level ≥ 5.5 mmol/L. [file 12882_2019_1611_MOESM1_ESM.tif]

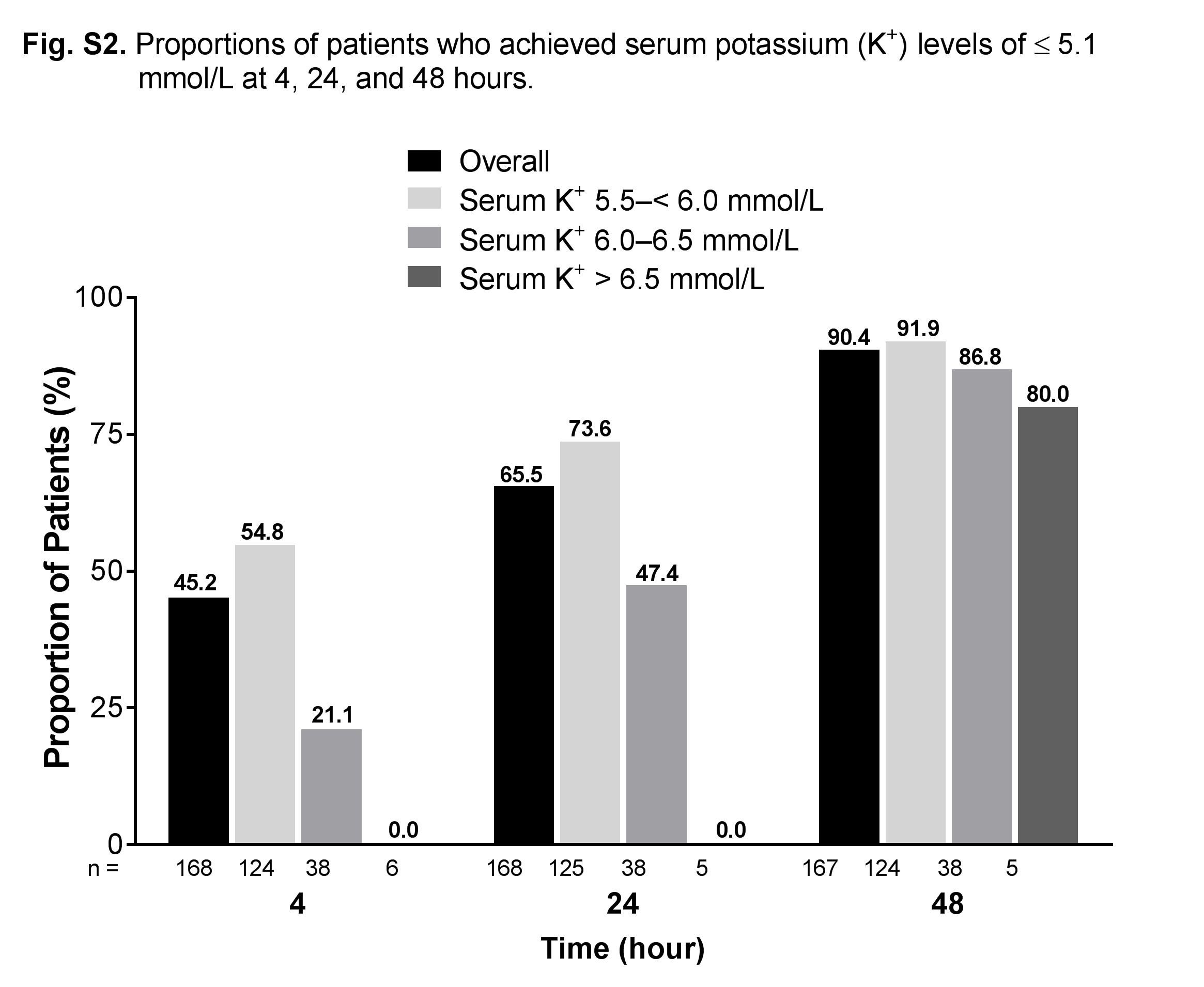

Supplement: Supplementary file 2 — Additional file 2: Figure S2. Proportions of patients who achieved serum potassium (K+) levels of ≤ 5.1 mmol/L at 4, 24, and 48 h. [file 12882_2019_1611_MOESM2_ESM.tif]

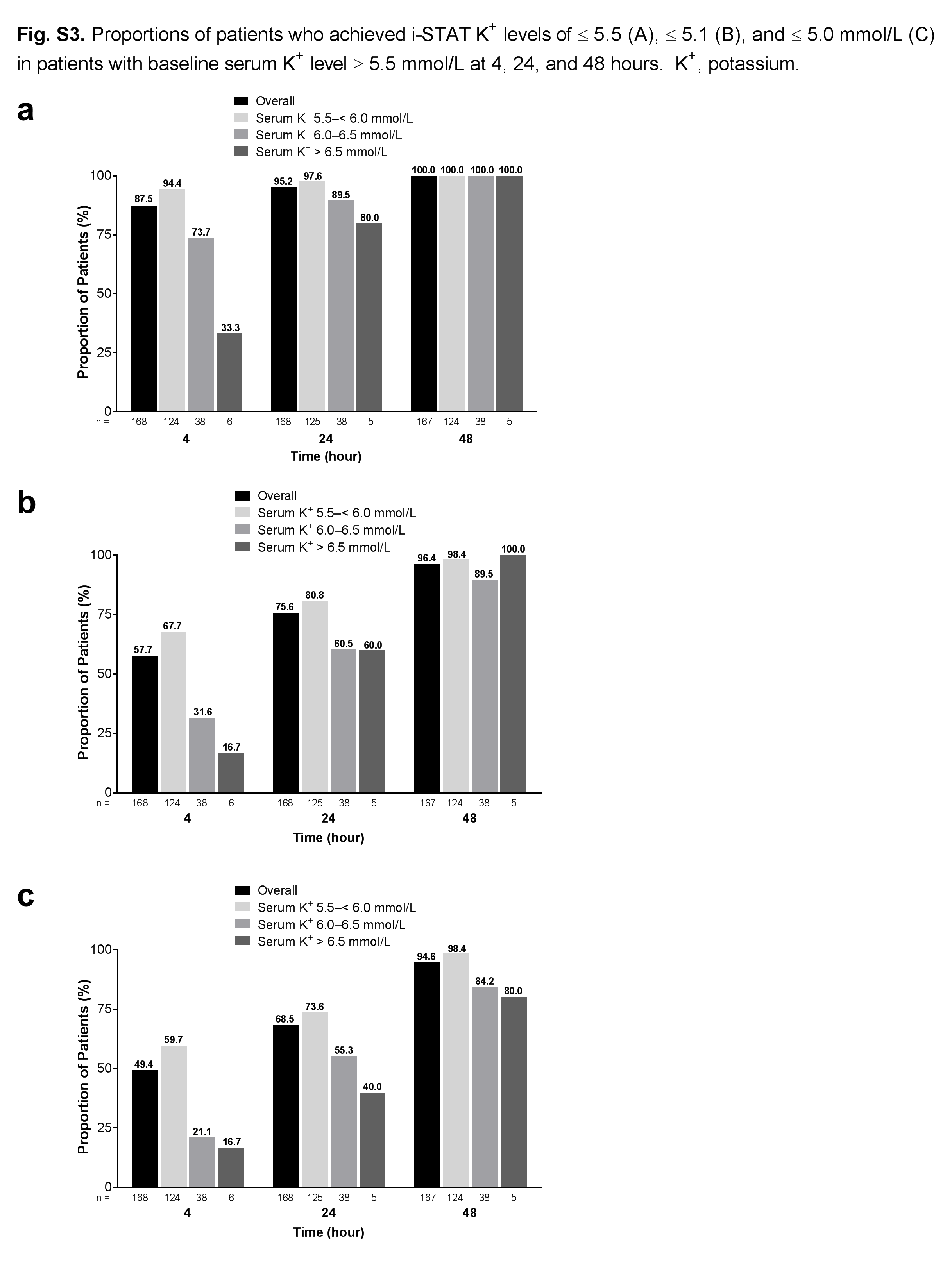

Supplement: Supplementary file 3 — Additional file 3: Figure S3. Proportions of patients who achieved i-STAT K+ levels of ≤ 5.5 (A), ≤ 5.1 (B), and ≤ 5.0 mmol/L (C) in patients with baseline serum K+ level ≥ 5.5 mmol/L at 4, 24, and 48 h. [file 12882_2019_1611_MOESM3_ESM.tif]
