# Supplementary material for: Efficacy and safety of sodium zirconium cyclosilicate in patients with baseline serum potassium level ≥ 5.5 mmol/L: pooled analysis from two phase 3 trials
Source: BMC Nephrol. 2019 Dec 2;20:440. doi: 10.1186/s12882-019-1611-8 (PMC6889520; doi:10.1186/s12882-019-1611-8)
Supplement: Supplementary file 4 — Additional file 4: Table S1. Median time to achievement of serum K+ level ≤ 5.1 mmol/L in the correction phase. [file 12882_2019_1611_MOESM4_ESM.docx]

**Supplementary Table S1**

Median time to achievement of serum K^+^ level ≤ 5.1 mmol/L in the correction phase.

| **Median (95% CI) time to serum K^+^ level, hours** | **Overall**  **(N = 170)** | **Baseline Serum K^+^ level (mmol/L)** | | |
| --- | --- | --- | --- | --- |
|  |  | **5.5–< 6.0**  **(n = 125)** | **6.0–6.5**  **(n = 39)** | **> 6.5**  **(n = 6)** |
| Serum K^+^ ≤ 5.1 mmol/L | 4.10  (3.95–20.93) | 4.00  (3.80–4.22) | 21.83  (4.00–23.63) | 45.30  (1.00–48.70) |

CI, confidence interval; K^+^, potassium.
