# Supplementary material for: Efficacy and safety of sodium zirconium cyclosilicate in patients with baseline serum potassium level ≥ 5.5 mmol/L: pooled analysis from two phase 3 trials
Source: BMC Nephrol. 2019 Dec 2;20:440. doi: 10.1186/s12882-019-1611-8 (PMC6889520; doi:10.1186/s12882-019-1611-8)
Supplement: Supplementary file 5 — Additional file 5: Table S2. Median time to achievement of i-STAT K+ levels ≤ 5.5, ≤ 5.1, and ≤ 5.0 mmol/L in patients with baseline serum K+ level ≥ 5.5 mmol/L in the correction phase. [file 12882_2019_1611_MOESM5_ESM.docx]

**Supplementary Table S2**

Median time to achievement of i-STAT K^+^ levels ≤ 5.5, ≤ 5.1, and ≤ 5.0 mmol/L in patients with baseline serum K^+^ level ≥ 5.5 mmol/L in the correction phase.

| **Median (95% CI) time to i‑STAT K^+^ level, hours** | **Overall**  **(N = 170)** | **Baseline Serum K^+^ level (mmol/L)** | | |
| --- | --- | --- | --- | --- |
|  |  | **5.5–< 6.0**  **(n = 125)** | **6.0–6.5**  **(n = 39)** | **> 6.5**  **(n = 6)** |
| i-STAT K^+^ ≤ 5.5 mmol/L | 1.06  (1.02–1.75) | 1.02 (1.00–1.07) | 2.00 (1.10–3.97) | 13.19 (1.00–142.52) |
| i-STAT K^+^ ≤ 5.1 mmol/L | 3.83  (2.05–4.00) | 2.08  (2.00–3.93) | 19.63  (3.77–22.22) | 34.26  (2.07–142.52) |
| i-STAT K^+^ ≤ 5.0 mmol/L | 4.00  (3.92–5.08) | 3.95  (2.15–4.00) | 21.83  (4.00–23.63) | 46.56  (4.00–216.33) |

CI, confidence interval; K^+^, potassium.
