# Supplementary material for: Efficacy and safety of sodium zirconium cyclosilicate in patients with baseline serum potassium level ≥ 5.5 mmol/L: pooled analysis from two phase 3 trials
Source: BMC Nephrol. 2019 Dec 2;20:440. doi: 10.1186/s12882-019-1611-8 (PMC6889520; doi:10.1186/s12882-019-1611-8)
Supplement: Supplementary file 6 — Additional file 6: Table S3. Serum chemistry laboratory parameters in the correction phase (N = 166). [file 12882_2019_1611_MOESM6_ESM.docx]

**Table S3**

Serum chemistry laboratory parameters in the correction phase (N = 166).

| **Parameter** | **Baseline** | **48 hours** | **Nominal change from baseline** |
| --- | --- | --- | --- |
| Creatinine, mmol/L | 0.22  (0.20–0.24) | 0.21  (0.19–0.23) | –0.01*  (–0.01 to –0.01) |
| Glucose, mmol/L | 7.75  (7.10–8.40) | 7.30  (6.75–7.86) | –0.42  (–0.98 to 0.14) |
| eGFR, mL/min/1.73 m^2^ | 37.5  (33.5–41.4) | 39.6  (35.4–43.7) | 2.2*  (1.3–3.2) |
| BUN, mmol/L | 15.44  (14.19–16.71) | 14.4  (13.19–15.61) | –1.04*  (–1.34 to –0.75) |
| Bicarbonate, mmol/L | 21.6  (20.9–22.3) | 23.1  (22.5–23.7) | 1.6*  (1.2–1.9) |
| Calcium, mmol/L | 2.35  (2.32–2.38) | 2.26  (2.24–2.29) | –0.08*  (–0.10 to –0.07) |
| Magnesium, mmol/L | 0.81  (0.79–0.84) | 0.79  (0.77–0.81) | –0.02*  (–0.03 to –0.02) |
| Sodium, mmol/L | 138.5  (138.0–139.0) | 139.5  (139.1–140.0) | 1.0*  (0.6–1.4) |

Data are shown as mean (95% confidence interval).
**p* ≤ 0.001 for change from baseline.

BUN, blood urea nitrogen; eGFR, estimated glomerular filtration rate.
